# Robust Peak Recognition in Intracranial Pressure Signals

**DOI:** 10.1186/1475-925X-9-61

**Published:** 2010-10-19

**Authors:** Fabien Scalzo, Shadnaz Asgari, Sunghan Kim, Marvin Bergsneider, Xiao Hu

**Affiliations:** 1 Department of Neurosurgery, Geffen School of Medicine, Neural Systems and Dynamic Lab (NSDL), University of California, Los Angeles, CA, USA

## Abstract

**Background:**

The waveform morphology of intracranial pressure pulses (ICP) is an essential indicator for monitoring, and forecasting critical intracranial and cerebrovascular pathophysiological variations. While current ICP pulse analysis frameworks offer satisfying results on most of the pulses, we observed that the performance of several of them deteriorates significantly on abnormal, or simply more challenging pulses.

**Methods:**

This paper provides two contributions to this problem. First, it introduces MOCAIP++, a generic ICP pulse processing framework that generalizes MOCAIP (Morphological Clustering and Analysis of ICP Pulse). Its strength is to integrate several peak recognition methods to describe ICP morphology, and to exploit different ICP features to improve peak recognition. Second, it investigates the effect of incorporating, automatically identified, challenging pulses into the training set of peak recognition models.

**Results:**

Experiments on a large dataset of ICP signals, as well as on a representative collection of sampled challenging ICP pulses, demonstrate that both contributions are complementary and significantly improve peak recognition performance in clinical conditions.

**Conclusion:**

The proposed framework allows to extract more reliable statistics about the ICP waveform morphology on challenging pulses to investigate the predictive power of these pulses on the condition of the patient.

## 1 Background

Traumatic Brain Injuries (TBI) affect more than 2 million people annually in the United States, and their incidence in the world keeps increasing [1]. The treatment of TBI patients in critical care units, as well as other neurological disorders, relies on the continuous measurement of intracranial pressure (ICP) (*i.e*. the sum of the pressures exerted within the craniospinal axis system). It is known that the management of ICP can attenuate secondary brain injuries and improve chances of recovery. Interestingly, the morphology of ICP waveform holds essential informations about the intracranial adaptive capacity (elastance), and even the outcome of head injured patients [2,3]. For example, it has been shown that variations of the ICP pulse morphology are linked to the development of intracranial hypertension [4-6], cerebral vasospasm [7], changes in the cerebral blood carbon dioxide (CO2) levels [8,9], decreased cerebral bloob flow (CBF) [10], and changes in the craniospinal compliance [11].

**Figure 1:**
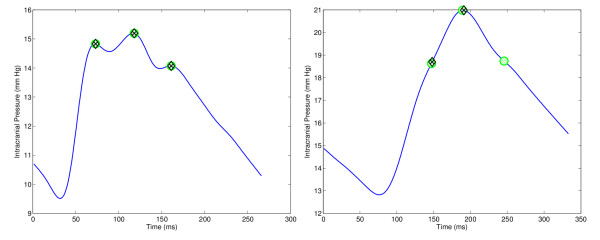
**Illustration of two ICP pulses (the actual position of the peak is depicted in green, MOCAIP prediction in black)**. On the left, an ICP dominant pulse is correctly annotated with the position of the three peaks. On the right, the automatic annotation failed to correctly recognized the third peak because of the uncommon shape of the pulse. This pulse is considered as a challenging one in our study.

The extraction of morphological features is essential to monitor and to understand ICP in an automatic fashion with the ultimate goal of improving the treatment of pathophysiological intracranial and cerebrovascular conditions. Although ICP pulses are typically triphasic [8] (*i.e*. three peaks), their shape can exhibit irregular variations such that some peaks may be missing. The recognition of these top peaks is a challenging task that has recently drawn special attention from different research groups. Several algorithms have been developed to detect the first peak [12], and to recognize the three peaks of ICP pulses [13-17]. Existing methods can be divided into two categories depending if they work offline, like Morphologram [14], or online, like MOCAIP [13,15] (**Mo**rphological **C**lustering and **A**nalysis of **I**CP **P**ulse). These techniques offer a satisfactory accuracy to recognize the peaks in general cases. However our recent observations show that their performance deteriorates significantly when the pulses exhibit abnormalities or are simply more challenging (a pulse is considered to be challenging if any of its peaks fails to be correctly designated by the baseline MOCAIP algorithm [15], see Figure 1). Such ICP pulses are of particular interest because we suspect that they might hold essential predictive information about the patient condition.

This paper investigates how to improve peak recognition accuracy on challenging ICP pulses. The contribution is two-fold. First, to conduct this study, MOCAIP++, a robust ICP pulse processing framework that generalizes MOCAIP, is introduced. The strength of MOCAIP++ relies on its capacity to integrate different peak recognition methods, and to exploit additional features based on the derivatives of the ICP signal. Our experiments evaluate these characteristics by providing a comparative analysis of three different state-of-the-art peak recognition techniques based on Gaussian Models, Gaussian Mixture Model (GMM), and Spectral Regression Analysis (SR), and by evaluating the impact of ICP features, such as curvature, first and second derivatives on the recognition performance. Second, this paper investigates the effect of incorporating challenging pulses into the training set of peak recognition methods learned in a supervised way. A method is proposed to sample automatically a representative challenging dataset of ICP pulses from a large database of ICP signals collected from 128 neurosurgical patients. The original, and the challenging datasets allow to study how the performance of peak recognition methods, essential to extract morphological features, can be improved.

## 2 Methods

### 2.1 ICP Dataset

Generally, ICP signal recordings consist of several hours long segments. By reviewing those files, we observed that the majority of the recordings contain pulses whose peaks are easily recognized by automatic algorithms. A subset of ICP files, however, contains pulses that are not correctly annotated by automatic peak recognition methods. One reason for these mismatches is that the pulse morphology differs significantly from the most common ones. We consider those pulses to be challenging (an example is shown in Figure 1).

The variation in morphology of these challenging pulses might originate from a combination of external factors such as sampling rate of the ICP, noise and artifact due to the acquisition device, or coughing of the patient. It is also possible that some of these morphological variations come from the condition of the patient and that they might hold relevant predictive information. Unfortunately, the peak recognition accuracy of current techniques on the ICP recordings containing those pulses drops dramatically. It is no longer possible to extract reliable statistics about their ICP waveform morphology to perform further analysis. This observation led us to extract a challenging dataset *D*' (Section 2.1.2) from the dataset *D* (Section 2.1.1). The new dataset *D*' is sampled from the recordings of *D* that contain a large percentage of challenging pulses. Both datasets, that are described below, will be used in the experiments to evaluate the performance of our framework. In addition, we will investigate if the use of the challenging dataset as part of the training set of peak recognition methods can improve their performance.

#### 2.1.1 Original Data

The source dataset of ICP signals originates from patients admitted to the University of California Los Angeles (UCLA) medical center. Its usage in the present study was approved by the UCLA Internal Review Board. It is a large, representative dataset that is reasonably distributed across gender, age, and type of patient (ICU or NON-ICU). A small portion of this dataset was previously used to evaluate MOCAIP [15] and its extensions based on regression analysis [18]. The ICP and ECG signals were acquired from 128 patients treated for various intracranial pressure relted conditions. ICP was monitored continuously using Codman intraparenchymal microsensors (Codman and Schurtleff, Raynaud, MA) placed in the right frontal lobe. ICP signals were recorded from bedside monitors using corporate data acquisition systems at a sampling frequency of either 240 Hz or 400 Hz. A total of 1425 recordings were extracted, each totalizing several hours. Those ICP and ECG signal recordings were subsequently pre-processed by MOCAIP so that they were first divided into 3 minutes segments. Then, a hierarchical clustering was applied on individual pulses of each segment, and the center of the dominant cluster was extracted to produce a dominant pulse. This clustering process leads to a representative set of 87,125 dominant pulses. It is referred to as the original dataset *D* from which a smaller, but more challenging dataset will be sampled. The actual positions of the three peaks in the ICP are obtained by manual annotation from experienced researchers following the procedure described in the next subsection.

#### 2.1.2 Challenging Data

The selection of a challenging subset of ICP pulses *D*' ⊂ *D* is achieved using a weighted sampling procedure from the file recordings of the original dataset *D*. Intuitively, the sampling aims at extracting more pulses from recordings that contain a larger percentage of challenging pulses so that they are better represented in the resulting dataset. To do so, each recording is associated with a weight corresponding to its degree of difficulty which is high if MOCAIP often fails to recognize the three peaks. The procedure to weight the files is described below.

Experienced researchers establish the groundtruth by manually setting the positions of the three peaks (*p*_1_, *p*_2_, and *p*_3_) in each pulse. The task of the researcher is to pick the right peak candidates among those automatically detected at curve inflections (Section 2.2.2). Whenever one of the three peaks is missing, its position is labelled with the empty set. Among the set of pulses, 7173 have missing *p*_1_, 3699 have missing *p*_2_, and 4626 have missing *p*_3_. Researchers cross-validate their results and, if necessary, they harmonize them using the annotation of the previous and following pulses as reference. For a few difficult cases where the researchers could not agree on the position of some peaks, the pulse was removed from the dataset. This procedure ensures that the groundtruth is not biased to a specific researcher.

**Figure 2:**
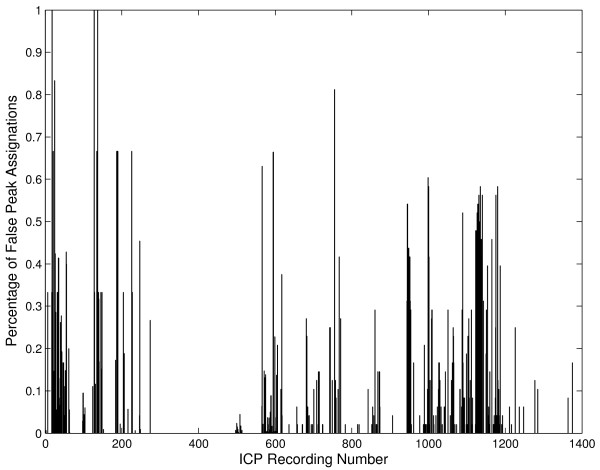
**The distribution of the weights is illustrated for the files in the original dataset of ICP pulses**. The weight of each file is set proportional to the percentage of wrong peak assignations. For example, a value of 1 indicates that all the peaks in that file were not assigned correctly by the MOCAIP, this usually happens in short recordings.

In parallel, MOCAIP is applied to annotate each pulse with the position of the three peaks (*p*_1_, *p*_2_, and *p*_3_). To find difficult files, the predictions of MOCAIP are compared with the manual groundtruth. For each ICP file *f_i_*_= 1...*F*_, a weight *w_i_*_= 1...*F *_is set proportional to the percentage of wrongly assigned peaks. This is done by comparing the position of each peak of the ground truth to the position obtained from the automatic files,

(1) $$w_{i}=\frac{{ℰ}_{p1}+{ℰ}_{p2}+{ℰ}_{p3}}{N_{p1}+N_{p2}+N_{p3}},$$

where ℰ_*p*1_, ℰ_*p*2_, ℰ_*p*3 _are the number of wrongly assigned peaks and $N_{p}{}_{\text{1}},N_{p}{}_{\text{2}},N_{p}{}_{\text{3}}$ are the total number of occurrences in the file of the peaks *p*_1_, *p*_2_, and *p*_3_, respectively. The distribution of the weights is illustrated in Figure 2.

Finally, the challenging dataset *D*' is created by extracting pulses using weighted sampling, such that a pulse has a probability *v_i _*(Eq. 2) to be picked from file *f_i_*. Therefore, files with large probability *v_i _*will contribute to more pulses in the sampled dataset.

(2) $$v_{i}=\frac{w_{i}}{\sum_{i=1}^{F}w_{i}}$$

To avoid redundancy from the files that contain only a few pulses, each pulse is selected at most once during sampling. The resulting dataset is made of 10638 ICP pulses among which 2816 have missing *p*_1_, 604 have missing *p*_2_, and 692 have missing *p*_3_. The challenging pulses are distributed among 58 patients.

### 2.2 MOCAIP++

This section introduces MOCAIP++, a generalization of the recently developed MOCAIP [15] which is an end-to-end framework that processes raw ICP signals to extract morphological waveform features through the recognition of the three peaks of the pulse. In its original form, MOCAIP relies on a Gaussian model to represent the prior knowledge about the position of each peak in the pulse. The Gaussian priors were replaced by a regression model in a recent extension [18].

**Figure 3:**
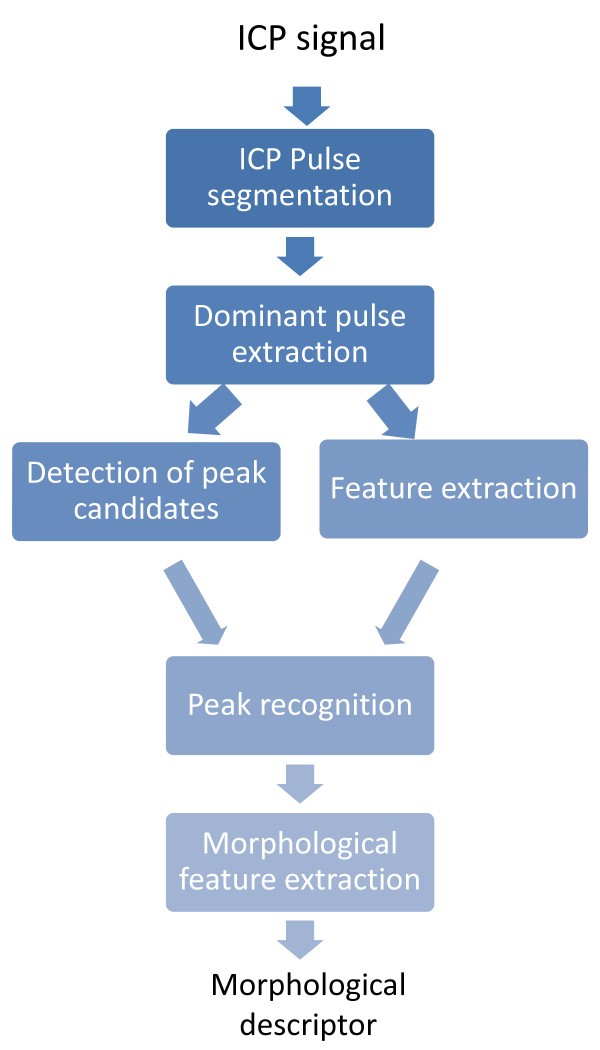
**Diagram showing the different modules in MOCAIP++ framework**.

MOCAIP++ generalizes its predecessors in two ways. First, it proposes a unifying view such that different peak recognition techniques can be integrated within the framework. Second, an additional processing step allows to exploit ICP features regardless the peak recognition method that is used. Similarly to MOCAIP, a pulse extraction technique (Section 2.2.1) first process the ICP signal to extract a reliable dominant pulse from which peak candidates are located at curves inflections (Section 2.2.2). Then, MOCAIP++ extracts different ICP features from the dominant pulse (such as curvature, first, and second derivative) (Section 2.2.3). The peak recognition module (Section 2.2.4) exploits the peak candidates and the features to recognize the peaks within the pulse. Finally, various statistics are estimated using the latency of these peaks and their ICP elevation (additional details can be found in the original papers [15,18]). The core of the algorithm is illustrated in Figure 3 and its major components are described in the next subsections.

#### 2.2.1 ICP Segmentation and Dominant Pulse Extraction

The first component of the framework (ICP pulse segmentation) takes a raw, continuous ICP signal and splits it in a series of individual ICP pulses. An individual pulse is found using a pulse extraction technique [19] combined with the ECG QRS detection [20] that locates each ECG beat. Therefore, the latency of the three peaks within the ICP pulse is relative to the ECG QRS.

Because ICP recordings are subject to various noise and artifacts during the acquisition process, a robust dominant pulse *S_i _*is extracted from a sequence of consecutive ICP pulses using hierarchical clustering [21]. It corresponds to the centroid of the largest cluster. In other words, the dominant pulse summarizes a short segment of consecutive ICP pulses.

#### 2.2.2 Detecting Peak Candidates

Then, MOCAIP++ detects peak candidates (*a*_1_, *a*_2_, ..., *a_N_*) at curve inflections of the dominant ICP pulse *S_i _*by segmenting the pulse into concave and convex regions using the second derivative of the signal. A peak is said to occur at the intersection of a convex and a concave region on a rising edge of ICP pulse, or at the intersection of a concave and a convex region on the descending edge of the pulse.

#### 2.2.3 ICP Features

**Figure 4:**
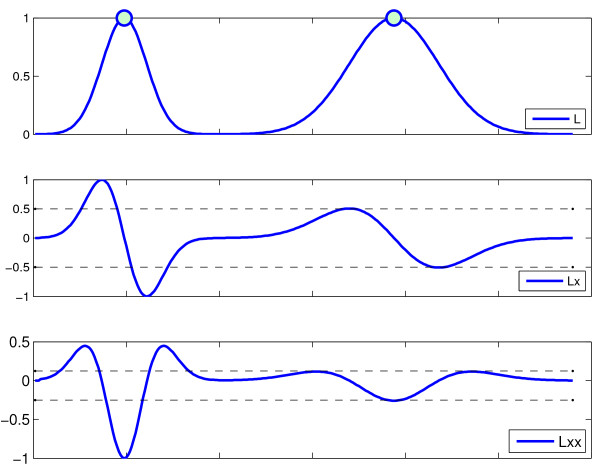
**Signal *L *made of two Gaussian peaks with different standard deviations**. Its first *L_x _*and second *L_xx _*derivatives are particularly usefull to discriminate peaks because their amplitude depends on the peak width but remains invariant to any global shift in elevation of the signal.

Previous MOCAIP-based studies [15,18] exploited the dominant pulses directly as input to peak recognition techniques. In signal processing, it is common to derive features that emphasize different properties of the signal. For example, the first derivative measures the changing rate of the signal with respect to time. As illustrated in Figure 4, it is particularly interesting in our case because for a similar amplitude, a wide peak, and a narrow peak will lead to different derivative values. Therefore, features extracted from the ICP signal derivative provide additional morphological characteristics that should help to discriminate between ICP peaks. One advantage of using these features is that they are invariant to a shift of the signal elevation. Note that the framework is not restricted to these features, any other features could in principle be exploited. In our experiments, we will evaluate the impact of using the first *L_x _*and second *L_xx _*derivatives, as well as the curvature *K *extracted from the ICP signal within MOCAIP++ framework.

##### First Derivative

For more robustness, the ICP signal *I*(*x*) is first convolved with a Gaussian smoothing filter G(x;σ) where σ is the standard deviation of the Gaussian (σ = 3 in our experiments),

(3) L(x,σ)=G(x;σ)*I(x).

Then the derivative *L_x _*is computed according to the smoothed version *L *of the ICP,

(4) $$L_{x}=L(x,\sigma )–L(x+\text{1},\sigma ).$$

##### Second Derivative

Similarly, the computation of the second derivative *L_xx _*relies on the first derivative *L_x_*,

(5) $$L_{xx}=L_{x}(x,\sigma )–L_{x}(x+1,\sigma ).$$

##### Curvature

The curvature *K *is computed as a ratio between the first and the second derivative of the signal,

(6) $$K=\frac{L_{xx}}{{\left(1+L_{x}^{2}\right)}^{3/2}}.$$

#### 2.2.4 Peak Recognition

This module aims at recognizing the three peaks (*p*_1_, *p*_2_, *p*_3_) within an ICP pulse among the set of candidate peaks (*a*_1_, *a*_2_, ..., *a_N_*). Depending on the recognition technique, it can exploit the latency of the peak candidates, the raw ICP pulse, or different features extracted from the pulse. In the next, we describe three different peak recognition approaches. They are based on independent Gaussian models [15], Gaussian Mixture Models (GMM), and Spectral Regression (SR) analysis [18], respectively.

##### (a) Gaussian Model

The original MOCAIP algorithm exploits Gaussian priors to identify the most likely configuration of the three peaks among the set of candidates. Given *P*(*X*_1_), *P*(*X*_2_), and *P*(*X*_3_) to denote the Gaussian probability distribution of the prior position of the three peaks (*p*_1_, *p*_2_, *p*_3_), peak recognition amounts to searching for the maximum of the following objective function,

(7) J(x,y,z)=P(X1=ax)+P(X2=ay)+P(X3=az)| ax∈a∧ay∈a∧az∈a,| x<y<z,

where *P*(*X_i _*= *a_k_*) represents the probability of assigning *a_k _*to the *i*-*th *peak.

In order to deal with missing peaks, an empty designation *a*_0 _is added to the pool of candidates. In addition, to avoid false designation, MOCAIP uses a threshold ρ such that *P*(*X_i _*= *a_k_*) = 0, *i *∈ {1, 2, 3}, *k *∈ {1, 2, ..., *N*} if the probability of assigning *a_k _*to *p_i _*is less than *ρ*.

##### (b) Gaussian Mixture Models

In contrast with MOCAIP that uses a model of independent Gaussian distributions to represent the likely position of each peak, the method proposed in this paragraph exploits a multi-modal distribution to model the joint latency of the three peaks. Observed peak configurations are approximated by a Gaussian Mixture Model (GMM), where each component *i *represents a cluster of configurations *μ_i _*of the three peaks. A GMM is defined as,

(8) $$P(X=x|\Theta )=\sum_{i=1}^{C}\alpha_{i}G(x;\mu_{i},\Sigma_{i}),$$

(9) $$G(x;\mu_{i},\Sigma_{i})=\frac{1}{\sqrt{2\pi \Sigma_{i}^{2}}}\exp^{\frac{-{\left(x-\mu_{i}\right)}^{2}}{2\Sigma_{i}^{2}}},$$

where α*_i_*, μ*_i_*, ∑_*i *_are the relative weight, the mean, and the variance of an individual component *i*, and *C *is the total number of components. For learning, Expectation-Maximization (EM) was used to estimate the model parameters θ = (*α*_1...*C *_; μ_1...*C *_; ∑_1...*C*_) that maximizes the likelihood of the observed peak configurations. EM was performed for a different number of components *C *∈ {1, ..., 10}. The number which minimizes the Bayesian Information Criterion (BIC) [22] was selected.

The detection task amounts to find the best configuration of the three peaks among the set of peak candidates *a *= (*a*_1_, *a*_2_, ..., *a_N_*) detected in the current pulse. This can be done by finding the configuration that is the more likely on the GMM,

(10) {p1,p2,p3}=argmaxp˜1,p˜2,p˜3P(X={p˜1,p˜2,p˜3}|Θ) | p˜1∈a∧p˜2∈a∧p˜3∈a| p˜1<p˜2<p˜3.

However, an additional difficulty is caused by missing peaks. One way to solve this problem is to use a hierarchical recognition approach where the possible configurations are first evaluated on the 3 peak model. If the largest response *r*_123 _= *P*(*X *= {*p*_1_, *p*_2_, *p*_3_}|Θ) fails to be above a given threshold τ_3_, the marginals *X*_12_, *X*_13_, *X*_23 _using only two dimensions of the model are evaluated,

(11) $$\{p_{1},p_{2}\}=\underset{{\tilde{p}}_{1},{\tilde{p}}_{2}}{\arg\max}P(X_{12}=\{{\tilde{p}}_{1},{\tilde{p}}_{2}\}|\Theta )\;|\;{\tilde{p}}_{1}\in a\wedge {\tilde{p}}_{2}\in a,{\tilde{p}}_{1}\neq {\tilde{p}}_{2},$$

(12) $$\{p_{1},p_{3}\}=\underset{{\tilde{p}}_{1},{\tilde{p}}_{3}}{\arg\max}P(X_{13}=\{{\tilde{p}}_{1},{\tilde{p}}_{3}\}|\Theta )\;|\;{\tilde{p}}_{1}\in a\wedge {\tilde{p}}_{3}\in a,{\tilde{p}}_{1}\neq {\tilde{p}}_{3},$$

(13) $$\{p_{2},p_{3}\}=\underset{{\tilde{p}}_{2},{\tilde{p}}_{3}}{\arg\max}P(X_{23}=\{{\tilde{p}}_{2},{\tilde{p}}_{3}\}|\Theta )\;|\;{\tilde{p}}_{2}\in a\wedge {\tilde{p}}_{3}\in a,{\tilde{p}}_{2}\neq {\tilde{p}}_{3},$$

and *r*_12 _= *P*(*X*_12 _= {*p*_1_, *p*_2_}|Θ), *r*_13 _= *P*(*X*_13 _= {*p*_1_, *p*_3_}|Θ), *r*_23 _= *P*(*X*_23 _= {*p*_2_, *p*_3_}|Θ). Again, if the maximum response to the GMM model of all the 2-peak configurations max(*r*_12_, *r*_13_, *r*_23_) is below a certain threshold *τ*_2_, 1-peak marginals *X*_1_, *X*_2_, *X*_3 _are evaluated, and the peak with the maximum response is marked.

(14) $$p_{1}=\underset{{\tilde{p}}_{1}}{\arg\;\max}P(X_{1}=\{{\tilde{p}}_{1}\}|\Theta )\;|\;{\tilde{p}}_{1}\in a$$

(15) $$p_{2}=\underset{{\tilde{p}}_{2}}{\arg\;\max}P(X_{2}=\{{\tilde{p}}_{2}\}|\Theta )\;|\;{\tilde{p}}_{2}\in a$$

(16) $$p_{3}=\underset{{\tilde{p}}_{3}}{\arg\;\max}P(X_{3}=\{{\tilde{p}}_{3}\}|\Theta )\;|\;{\tilde{p}}_{3}\in a$$

##### (c) Spectral Regression

In a recent comparison of regression techniques [18], Spectral Regression (SR) [23] demonstrated excellent accuracy in peak recognition on standard ICP pulses. This motivates us to select it as the baseline regression method within MOCAIP++. The regression model *y_i _*= *f*(*x_i_*) maps the position of the peaks as a function of the ICP dominant pulse. The model is automatically learned from training ICP pulses *S *= {*S_i _*= 1...*n*} labeled with the latency of the peaks *y_i _*= (*p*_1_, *p*_2_, *p*_3_) within the pulse. Each pulse *S_i _*is resized to a vector *x_i _*∈ ℝ^*s *^of length *s *= 500 ms, and normalized in amplitude between [1].

SR combines spectral graph analysis and standard linear regression to obtain a model that gives similar predictions ${\hat{y}}_{i}\in \hat{Y}$ for data samples *x_i _*∈ *X *that are close (*i.e*. that are nearest neighbors in a graph representation), such that the following measure ϕ is minimized:

(17) $$\phi =\sum_{i,j=1}^{n}{\left({\hat{y}}_{i}-{\hat{y}}_{j}\right)}^{2}W_{i,j},$$

where *W *∈ ℝ^*n *× *n *^is the item-item similarity matrix that associates a positive value to *W_i,j _*if the samples *x_i_*, *x_j _*belong to the same class. This is done by first using the eigenvectors of the matrix *W*,

(18) We=λDe,

where *D *is a diagonal matrix whose entries are column sums of *W*, *D_i,i _*= Σ_*j *_*W_j,i_*, and *e*_0_, *e*_1_, ..., *e_d _*denote the *d *+ 1 eigenvectors with respect to the *d *+ 1 largest eigenvalues *λ*_0_≥*λ*_1_≥ ... ≥ *λ*_d_.

Then SR finds *d *vectors {${\hat{\beta }}_{0},{\hat{\beta }}_{1},\ldots ,\hat{\beta }$}that minimize the residual Sum of Square Error (SSE),

(19) $${\hat{\beta }}_{j}=\arg\min_{\beta }\sum_{i=1}^{n}{\left(\beta^{T}x_{i}-y_{i}^{j}\right)}^{2},$$

where $y_{i}^{j}$ is the *i*-th element of *e_j_*.

For recognition on a new pulse *x_j_*, the regression model *y_j _*= *f*(*x_j_*) predicts the most likely position of the three peaks *y_j _*= (*p*_1_, *p*_2_, *p*_3_). A nearest neighbor search is then performed so that the nearest candidate (*a*_1_, *a*_2_, ..., *a_N_*) to each prediction is assigned to the peak label corresponding to the matched prediction. Additional features *f_i _*∈ ℝ(^*s*^, where *f_i _*∈ {*L_x_*, *L_xx_*, *K*}, can be concatenated to the original input *x_i _*∈ ℝ(^*s *^to create a new input vector [*x_i _f_i_*] that combines both modalities.

Although Spectral Regression is a linear regression algorithm, it can easily be extended to become nonlinear by using a kernel projection (Radial Basis Function (RBF)) of the input vectors. We further refer to this technique as the Kernel Spectral Regression (KSR).

## 3 Results and Discussion

### 3.1 Accuracy of Peak Recognition Methods on Challenging Data

This section provides a comparative analysis of peak recognition techniques by evaluating their performance on the challenging dataset of ICP pulses. Models based on Gaussian (MOCAIP), Gaussian Mixtures (GMM), Spectral Regression (SR), and Kernel Spectral Regression (KSR) models are evaluated. A five-fold cross-validation is performed on the challenging dataset *D*', such that at each of the five iterations, four folds are used to train the model while the remaining one is retained for evaluation. The partitioning is randomly made with the constraint that the pulses of a given patient are grouped into the same fold. This ensures that data from the same patient are not present at the same time in the training and testing sets.

During evaluation, a predicted position ${\hat{y}}_{i}$_*i *_of one of the three peaks is considered to be correct if it is equal to the actual position *y_i _*established manually. Given that peaks may be set as missing in the groundtruth, True positive (TP), false positive (FP), true negative (TN), or false negative (FN) are defined as follow,

(20) $$\text{A prediticion }{\hat{y}}_{i}\text{ of }y_{i}\text{ is a}\{\begin{array}{ll} TP, & \text{if}({\hat{y}}_{i}=y_{i}\wedge {\hat{y}}_{i}\neq \emptyset \wedge y_{i}\neq \emptyset )\\ FP, & \text{if}({\hat{y}}_{i}\neq \emptyset \wedge y_{i}=\emptyset )\\ TN, & \text{if}({\hat{y}}_{i}=\emptyset \wedge y_{i}=\emptyset )\\ FN, & \text{if}(({\hat{y}}_{i}=\emptyset \vee ({\hat{y}}_{i}\neq y_{i}))\wedge y_{i}\neq \emptyset ) \end{array}$$

Based on these measures, the accuracy $A_{p}$ of one of the three peaks *p *∈ {*p*_1_, *p*_2_, *p*_3_} is defined as,

(21) $$A_{p}\text{=}\left(TP+TN\right)/\left(TP+FP+TN+FN\right).$$

**Figure 5:**
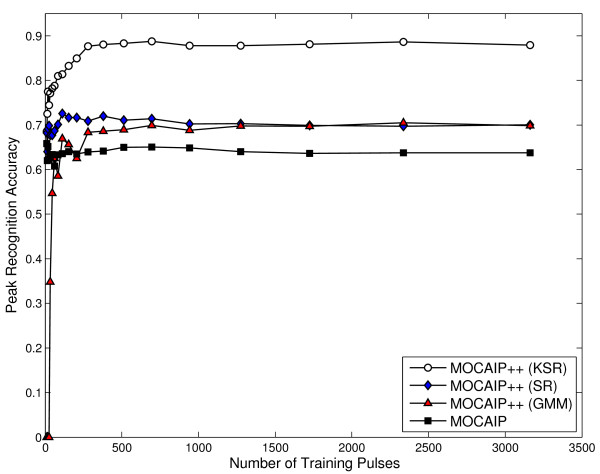
**Effect of the number of training samples on the average recognition accuracy (Eq. 21) for different models (KSR, SR, GMM, MOCAIP **[15]**) using a five-fold crossvalidation on our challenging dataset *D*'**. Results correspond to the average for the three peaks (*p*_1_, *p*_2_, and *p*_3_).

The accuracy $A_{p}$ of a peak *p *is obtained by averaging the accuracy over the five-folds. Similarly, the overall accuracy A is obtained by averaging the accuracy of the three peaks, $A=(A_{p_{1}}+A_{p_{2}}+A_{p_{3}})/3$. The learning of the recognition models is supervised in the sense that it relies on a set of manually labelled ICP pulses. As the number of training examples increases, the overall accuracy is generally expected to improve as well. We report this aspect by plotting the average prediction accuracy for each method against the number of training samples in Figure 5. To test one of the 5 folds, a model is trained by randomly extracting *n *pulses from the remaining 4 folds.

Results clearly indicates that KSR performs better by reaching a maximum accuracy of 88.78% ± 2.35, while the other techniques are less accurate; SR obtains 72.57 ± 2.6, GMM 70.47% ± 2.64, and MOCAIP 65.83% ± 2.96. It is interesting to notice that all the methods reaches their maximum accuracy before 500 training pulses. These results confirms that, besides KSR, current methods do not offer good recognition results on challenging pulses. Although KSR performs better than any other techniques, it requires all the training pulses to be kept as a part of the model to be able to compute the kernel projection. Nevertheless, KSR gives us an insight about what performance a peak recognition technique can achieve on our challenging dataset.

#### 3.1.1 Computational Cost

One of the possible applications our framework is to be used in portable devices to monitor ICP continuously. Such an application requires real time performances of the peak recognition techniques. This section evaluates the performance of the different recognition techniques in terms of their complexity by comparing their computational time during learning and recognition.

Table 1 shows that, on a dataset of 2000 ICP pulses, MOCAIP (Gaussian), and SR are the fastest for training their model with only 60 and 90 ms, while GMM is much more slower with 33,940 ms. For recognition on a single pulse, SR ranks first with 0.19 ms, MOCAIP second (6.7 times slower), GMM is 10 times slower, and KSR is about 100 times slower than SR. Batch recognition performance is measured on a set of 2000 pulses. Under these conditions, KSR improves a lot due to the optimization of matrix operations but remains behind SR. Note that the reported durations only represents the running time of the learning, and recognition methods. Additional time is needed for MOCAIP++ to pre-process the ICP, detect peak candidates, and to compute additional features such as curvature and signal derivatives. Running time were measured using built-in MATLAB functions. These tests were performed on a DELL OPTIPLEX 760 computer equipped with INTEL DUAL-CORE E8600 cadenced with a 3.33 GHz processor and 3 GB of RAM.

**Table 1:** Running time for learning peak recognition models (Gaussian, SR, KSR, and GMM) from 2000 ICP pulses, and for recognition on 1 and 2000 pulses.

	Gaussian	SR	KSR	GMM
Learning (2000 pulses)	70 ms	90 ms	1,340 ms	33,940 ms
	1.0	1.28	19	484

Recognition (1 pulse)	1.3 ms	0.19 ms	19.6 ms	2.3 ms
ratio	6.70	1.00	100.94	11.85

Recognition (2000 pulses)	2.861 sec	0.23 sec	2.028 sec	15.156 sec
ratio	12.39	1.00	8.79	65.64

While the Gaussian model performs the fastest for learning, SR-based model offers the best performance during recognition.

### 3.2 Feature-based Peak Recognition

This section evaluates the impact of the additional ICP features (Section 2.2.3) within MOCAIP++ on peak recognition performance. The same experimental protocol (five-fold cross-validation) of the previous section is used to evaluate the accuracy of SR, KSR, and GMM using three different features; curvature (Curv), first (*L_x_*) and second (*L_xx_*) derivatives on the challenging dataset *D*'.

**Figure 6:**
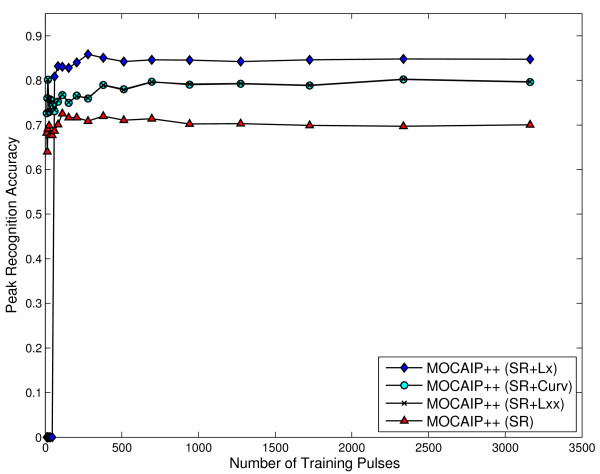
**The average recognition accuracy (Eq. 21) is reported versus the number of training samples**. Results illustrate the effect of three differential features on the SR model [18]: curvature (Curv), first (*L_x_*) and second (*L_xx_*) derivatives.

Figure 6 shows that each feature significantly improves the overall accuracy of the SR method. While the original SR-based recognition method [18] attains an accuracy of 72.57% ± 2.6, the use of the second derivative and curvature improves it to 80.26 ± 2.29 and 80.4% ± 2.2, respectively. SR performs best when it is combined with the first derivative *L_x _*of the ICP, reaching an accuracy of 85.81% ± 2.5. This constitutes a very significant result (+13%) in favor of our feature-based MOCAIP++ method.

**Figure 7:**
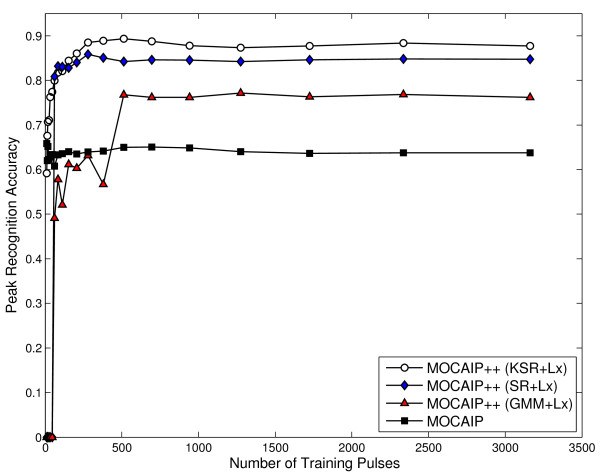
**Average recognition accuracy (Eq. 21) after a five-fold cross-validation for MOCAIP-based peak recognition methods improved with the use of the first derivative *L_x _*of the ICP**.

When combined with derivative-based features, GMM, and KSR methods exhibit a similar ranking of improvement; first derivative offers the largest effect on accuracy, while curvature and second derivatives generally have less significant improvement. With the use of the first derivative (see Figure 7), GMM method improves from 70.47% ± 2.64 to 77.14% ± 1.85, while KSR only shows a marginal improvement from 88.78% ± 2.35 to 89.36% ± 2.51. We have also noticed in additional experiments that combining different features, such as *L_x_*+*L_xx_*, does not improve the performance obtained by using only the first derivative *L_x _*of the ICP signal. These results demonstrate that the use of the first derivative within MOCAIP++ improves the recognition accuracy of the three peak recognition methods we have integrated. It can also be pointed out that the accuracy reached by *SR *+ *L_x _*is very close to *KSR *+ *L_x_*. Considering the previous remarks about the execution time and the storage of training samples for the kernel computation required for KSR, the use of SR combined with the first derivate seems to provide the right tradeoff between speed and accuracy for peak recognition on challenging ICP pulses.

### 3.3 Impact of the Training Data Sampling Strategy

Although peak recognition models are trained in a supervised fashion such that they integrate morphological information from pulses with known peaks into models that may correctly identify peaks in new pulses, the underlying training pulses affect the estimation of the parameters and the performance of such models. Intuitively, the model should be trained on a representative range of pulses (easy, or challenging) to gain sufficient precision. This section evaluates the effect of incorporating pulses extracted from the challenging dataset into the training set of peak recognition methods.

In these experiments, peak recognition methods are estimated from two different annotated training sets ($T_{1},T_{2}$). The first training set, named *reference library *$T_{1}$, is made of 3000 randomly selected ICP pulses from the original dataset *D*. These pulses present a wide range of morphological variations but the majority of them are generally easily annotated. A subset of these data was used in previous works [15] to train MOCAIP. The second training set, named *weighted sampling *$T_{2}$, is made of 1500 randomly selected pulses from the original dataset *D*, plus 1500 pulses randomly extracted from the challenging dataset *D*' created using a weighted sampling procedure (Section 2.1.2). Unlike previous section, where peak recognition methods were assessed against the challenging dataset *D*', the evaluation is now performed on the full dataset *D*. This allows us to see if the methods are not subject to overfitting; we verify if the methods that offer good results on challenging data also generalize well on regular pulses.

**Figure 8:**
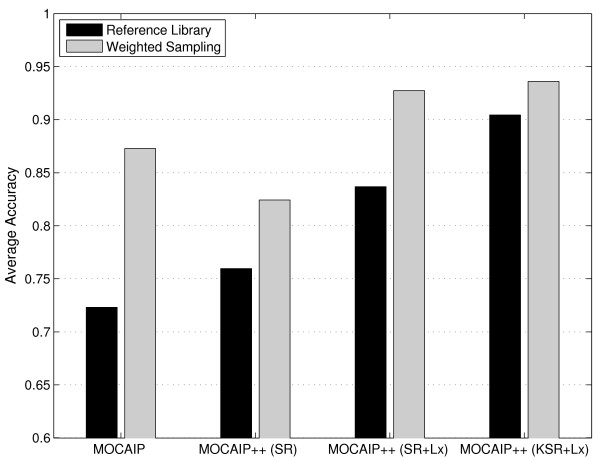
**Effect of the source data used to train the peak recognition models on the average recognition accuracy (Eq. 21) evaluated on the large dataset *D***. The reference library $T_{1}$ is made of randomly chosen ICP pulses. The second training set, weighted sampling$T_{2}$, is made of an equal number of randomly selected pulses from the large dataset *D* and challenging pulses randomly extracted from the challenging dataset *D*'. Both datasets contain 3000 pulses

Figure 8 gives the average accuracy. It can be seen that the use of an equal number of pulses sampled from the full and challenging dataset considerably improves the performance of peak recognition methods over models exclusively learned on random pulses. The improvement is as follows: MOCAIP, from 72.31% to 87.27%, SR, from 75.96% to 82.41%, SR+Lx, from 83.67% to 92.74%, and KSR+L_x _from 90.44% to 93.64%. The combination of our two contributions, the use of the first derivative and the weighted sampling for training, improves SR-based MOCAIP approach by about **17**% (from 75.96% to 92.74%). This is a very significant improvement of performance that should help to extract more reliable statistics about ICP pulse morphology in real clinical conditions.

## 4 Conclusions

Recent works suggest that changes in the waveform morphology of ICP may provide insight to forecasting critical intracranial and cerebrovascular pathophysiological variations. However, automatic analysis of the waveform morphology of ICP acquired in clinical conditions is still beyond current ICP analysis frameworks. Their performance deteriorates significantly when the morphology of the pulse exhibits uncommon morphological changes.

**Figure 9:**
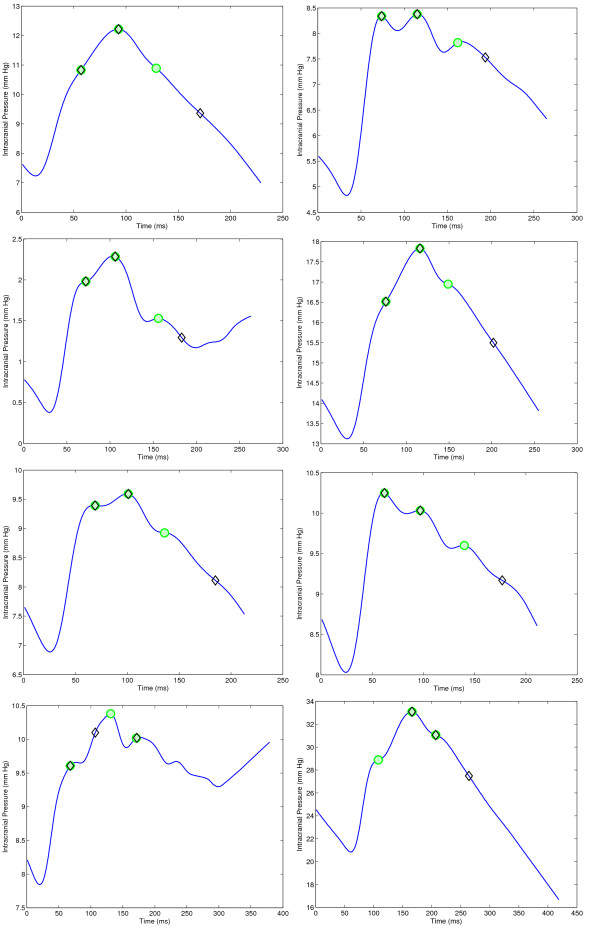
**Illustration of challenging ICP pulses where the original MOCAIP failed to recognize at least one of the peaks**. The actual position of the peaks, correctly predicted by MOCAIP++ (*SR *+ *L_x_*) are depicted by green circles, while the black diamonds correspond to the MOCAIP predictions

This paper has described MOCAIP++, a generalization of the recently developed MOCAIP, that provides a robust framework for analyzing Intracranial Pressure signal (ICP) in terms of its waveform morphology. The proposed approach improves current methods by allowing the integration of several peak recognition methods. In addition, whereas previous MOCAIP-based studies [15,18] exploited dominant pulses directly as input to peak recognition techniques, MOCAIP++ allows to derive additional features that capture more informative properties of the ICP signal and hence better discriminate the three peaks. The first derivative of the ICP signal has been shown to be the best among the features tested in our experiments (as shown in Figure 9). It improved all the peak recognition methods. This can be explained by its invariance to global shift in elevation from the baseline of the pulse. Performance in terms of peak recognition accuracy obtained by the proposed SR-based extension are close to the non-linear Kernel Spectral Regression (KSR). KSR can be considered really close to the best performing solution for this problem but it has the disadvantage to require to keep all the training samples, and is much slower than the SR.

Another contribution of this paper is to show that incorporating a challenging subset of ICP pulses into the training set of peak recognition methods has a positive effect on their overall accuracy.

Experiments on a large dataset of ICP signals, as well as on a representative collection of sampled challenging ICP pulses, demonstrate that both contributions are complementary and significantly improve the recognition performance of ICP peaks in real conditions. These findings provide insight in order to potentially improve other ICP peak recognition frameworks, and will help us to collect more reliable statistics about ICP morphology to further investigate its predictive power on patient condition.

## Competing interests

The authors declare that they have no competing interests.

## Authors' contributions

FS conceived the study, carried out the implementation of the framework, and drafted the manuscript. SA participated to the design of performance evaluation methods and statistical analysis. SK participated to the formalization of the framework. MB provided physiological and clinical background to the study. XH conceived the study including the idea of challenging pulse set and error-based sampling in addition to coordinating the execution of the study. All authors participated to the writing of the manuscript and approved the final manuscript.
